# AS160 deficiency causes whole-body insulin resistance via composite effects in multiple tissues

**DOI:** 10.1042/BJ20120702

**Published:** 2012-12-14

**Authors:** Hong Yu Wang, Serge Ducommun, Chao Quan, Bingxian Xie, Min Li, David H. Wasserman, Kei Sakamoto, Carol Mackintosh, Shuai Chen

**Affiliations:** *MOE Key Laboratory of Model Animal for Disease Study, Model Animal Research Center, Nanjing University, Pukou District, Nanjing 210061, China; †MRC Protein Phosphorylation Unit, College of Life Sciences, University of Dundee, Dundee DD1 5EH, Scotland, U.K.; ‡Nestlé Institute of Health Sciences SA, Campus EPFL, Quartier de l'Innovation, Bâtiment G, 1015 Lausanne, Switzerland; §Department of Molecular Physiology and Biophysics, Vanderbilt University, School of Medicine, 2200 Pierce Ave, Nashville, TN 37232, U.S.A.; ‖Division of Cell and Developmental Biology, College of Life Sciences, University of Dundee, Dundee DD1 5EH, Scotland, U.K.

**Keywords:** Akt substrate of 160 kDa (AS160), glucose transport, insulin resistance, liver, muscle, AS160, Akt substrate of 160 kDa, EDL, extensor digitorum longus, FBP-1, fructose-1,6-bisphosphatase 1, GAP, GTPase-activating protein, GAPDH, glyceraldehyde-3-phosphate dehydrogenase, GIR, glucose infusion rate, GLUT, glucose transporter, GSK3, glycogen synthase kinase 3, MBP, myelin basic protein, PCK/PEPCK, phosphoenolpyruvate carboxykinase, PKB, protein kinase B, PM, plasma membrane, RER, respiratory exchange ratio, TA, tibialis anterior

## Abstract

AS160 (Akt substrate of 160 kDa) is a Rab GTPase-activating protein implicated in insulin control of GLUT4 (glucose transporter 4) trafficking. In humans, a truncation mutation (R363X) in one allele of AS160 decreased the expression of the protein and caused severe postprandial hyperinsulinaemia during puberty. To complement the limited studies possible in humans, we generated an AS160-knockout mouse. In wild-type mice, AS160 expression is relatively high in adipose tissue and soleus muscle, low in EDL (extensor digitorum longus) muscle and detectable in liver only after enrichment. Despite having lower blood glucose levels under both fasted and random-fed conditions, the AS160-knockout mice exhibited insulin resistance in both muscle and liver in a euglycaemic clamp study. Consistent with this paradoxical phenotype, basal glucose uptake was higher in AS160-knockout primary adipocytes and normal in isolated soleus muscle, but their insulin-stimulated glucose uptake and overall GLUT4 levels were markedly decreased. In contrast, insulin-stimulated glucose uptake and GLUT4 levels were normal in EDL muscle. The liver also contributes to the AS160-knockout phenotype via hepatic insulin resistance, elevated hepatic expression of phosphoenolpyruvate carboxykinase isoforms and pyruvate intolerance, which are indicative of increased gluconeogenesis. Overall, as well as its catalytic function, AS160 influences expression of other proteins, and its loss deregulates basal and insulin-regulated glucose homoeostasis, not only in tissues that normally express AS160, but also by influencing liver function.

## INTRODUCTION

Insulin-regulated disposal of glucose from the bloodstream into skeletal muscle and fat tissues is mediated by the glucose transporter GLUT4, which undergoes translocation from intracellular storage sites on to the cell surface upon insulin stimulation [1]. The insulin-activated PI3K (phosphoinositide 3-kinase)/PKB (protein kinase B, also known as Akt) signalling axis plays a key role in mediating insulin-stimulated GLUT4 trafficking (reviewed in [2]). Hunting for substrate(s) downstream of PKB in controlling GLUT4 trafficking has been the focus of research in the field for decades. Thus far, the most convincing substrate is the Rab GAP (GTPase-activating protein) AS160 (Akt substrate of 160 kDa), that was first identified as a PKB/Akt substrate [3]. Insulin can promote phosphorylation of AS160 on multiple sites, including Ser^318^, Ser^341^, Ser^570^, Ser^588^, Thr^642^, Ser^666^ and Ser^751^ [3,4]. Subsequently, phosphorylated AS160 interacts with the 14-3-3 family of phosphoprotein-binding proteins, which is mainly mediated by phosphorylated Thr^642^ [4,5].

Knocking down AS160 in 3T3-L1 adipocytes increased the cell-surface expression of GLUT4 in the basal state [6,7], whereas overexpression of an AS160-4P mutant (in which Ser^318^, Ser^588^, Thr^642^ and Ser^751^ are substituted by non-phosphorylatable alanine) in both 3T3-L1 adipocytes [3] and L6 myocytes [8] could inhibit insulin-stimulated GLUT4 translocation on to the cell surface. Overexpression of the AS160-4P mutant in 3T3-L1 adipocytes impaired GLUT4 exocytosis, but not its endocytosis [9]. However, the exact site in GLUT4 exocytosis that AS160 affects has not yet been fully resolved. Although some data suggested that overexpression of the AS160-4P mutant affected exit of GLUT4 vesicles from the intracellular storage sites [9], other studies proposed that overexpression of the AS160-4P mutant affected the docking step of GLUT4 translocation in 3T3-L1 adipocytes [10,11]. When the AS160–14-3-3 interaction was disrupted through a point mutation in a knockin mouse (in which a non-phosphorylatable alanine residue replaces Thr^642^ on AS160), insulin-stimulated GLUT4 trafficking and glucose uptake was impaired in muscle, which consequently rendered the AS160 knockin mouse less sensitive to insulin [12]. These studies suggest that AS160 is involved in retaining GLUT4 within the cytosol, whereas AS160 phosphorylation and its binding to 14-3-3 is required for insulin to stimulate GLUT4 translocation on to the cell surface (reviewed in [13,14]). At least in the *in vitro* assay, AS160 possesses RabGAP activity towards downstream small G-protein Rabs 2A, 8A, 10 and 14 [15]. It has also been proposed that AS160 regulates GLUT4 trafficking through controlling Rab10 in 3T3-L1 adipocytes [16,17] and Rab8A in L6 myocytes [18].

Previously a premature stop mutation (R363X) on AS160 was identified in human patients with extreme postprandial hyperinsulinaemia [19]. Owing to limitations in studying humans, the effects of AS160 deficiency on glucose metabolism in adipose tissue and skeletal muscle have not been studied. Therefore we decided to generate an AS160-knockout mouse model to study how AS160 deficiency affects glucose metabolism.

## MATERIALS AND METHODS

### Materials

Recombinant human insulin was from Novo Nordisk and glucose was from Baxter Clintec. Collagenase (Type I) was from Worthington. Microcystin-LR was from Professor Linda Lawton (School of Life Sciences, Robert Gordon University, Aberdeen, U.K.), protease inhibitor cocktail tablets were from Roche Diagnostics and precast NuPAGE® Bis-Tris gels were from Invitrogen. Protein G–Sepharose was from GE Healthcare. 2-deoxy-D-[1,2-^3^H(N)]glucose and D-[1-^14^C]mannitol were from PerkinElmer. All other chemicals were from BDH Chemicals or Sigma–Aldrich.

### Antibodies

A rabbit antibody against the C-terminus of AS160 (catalogue number 07-741) was from Upstate/Millipore. A sheep antibody against the N-terminus (amino acids 1–280) of AS160 termed anti-AS160(N) [S149D, 3rd bleed, DSTT (Division of Signal Transduction Therapy)] was generated at the University of Dundee using GST (glutathione transferase)–AS160^1–280^ (human) fusion protein as the antigen, and column-purified against MBP (myelin basic protein)–AS160^1–280^. A sheep antibody against TBC1D1 was generated at the University of Dundee as described previously [20]. GLUT1 and GLUT4 antibodies were provided by Professor Geoffrey Holman (Department of Biology and Biochemistry, University of Bath, Bath, U.K.). Antibodies that recognize phosphorylated Ser^21^/Ser^9^ on GSK3α/β (glycogen synthase kinase 3α/β) (catalogue number 9331), phosphorylated Ser^473^ on PKB (catalogue number 9271), and anti-PKB (catalogue number 9272) and anti-PCK2 [recognizing PEPCK (phosphoenolpyruvate carboxykinase) 2] (catalogue number 6924) were from Cell Signaling Technology. Anti-GSK3α/β (catalogue number 44-610) was from Invitrogen. Anti-MBP (catalogue number ab9084) and anti-PCK1 (recognizing PEPCK1) (catalogue number ab87340) were from Abcam. Anti-FBP-1 (fructose-1,6-bisphosphatase 1) was from Santa Cruz Biotechnology (catalogue number sc-32435) and anti-GAPDH (glyceraldehyde-3-phosphate dehydrogenase) was from Sigma (catalogue number G8795).

### Generation of the AS160-knockout mouse

The AS160^T649A^ knockin mouse, in which the tenth exon harbouring the T649A mutation is flanked by loxP-Cre excision sites, was previously generated in our laboratory [12]. The AS160-knockout mouse was generated by mating the AS160^T649A^ knockin mouse with the Bal1 mouse line in which Cre recombinase is expressed in all tissues [21]. The tenth exon of AS160^T649A^ was excised in the resultant AS160-knockout mouse.

### Mouse breeding and genotyping

All animal studies, breeding and husbandry were approved by the Ethics Committees at the University of Dundee and Nanjing University. Mice were housed with a light/dark cycle of 12 h, and free access to food and water unless stated. Genotyping of AS160-knockout mice and wild-type littermates was performed by PCR using genomic DNA isolated from an ear biopsy as described previously [22]. Genotyping of the AS160-knockout allele was performed by PCR using the primers 5′-ATCTTGGGGCACTATCAACC-3′ and 5′-TAACCCGTCATACCTGACGAC-3′, and the wild-type allele was genotyped using the primers 5′-ATCTTGGGGCACTATCAACC-3′ and 5′-CAGTGGCATGATCTCTGTGG-3′.

### Tissue lysis and immunoprecipitation

Mouse tissues were lysed as described previously [22]. Protein concentrations were determined using Bradford reagent (Thermo Scientific).

Anti-AS160(N) antibody (1 μg of antibody/mg of tissue lysate protein) was used to immunoprecipitate full-length AS160 and truncated AS160^1–609^ from various mouse tissues. Briefly, tissue lysates were incubated with the antibody-coupled Protein G–Sepharose overnight at 4°C. After washing away non-specific-binding proteins, the immunoprecipitates were eluted in SDS sample buffer for subsequent analysis via Western blotting as described previously [20,23].

### Subcellular fractionation of adipose tissue

Subcellular fractionation was carried out as described previously [12]. Briefly, adipose tissue was homogenized in lysis buffer [22] with detergent omitted. The unbroken cells were removed from the homogenates by centrifugation at 500 ***g*** for 10 min. The supernatants were further centrifuged at 10000 ***g*** for 12 min to obtain crude PM (plasma membrane) together with nucleus and mitochondria, and an intracellular fraction containing cytoplasm and the GLUT4 storage vesicles. The PM fraction was solubilized in detergent-containing [1% (v/v) Triton X-100] lysis buffer.

### Western blot analysis and quantification

After separation by SDS/PAGE, proteins were immunoblotted on to nitrocellulose membranes that were blocked in milk and incubated at 4°C for 16 h using the antibodies indicated. Signals were detected using horseradish-peroxidase-conjugated secondary antibodies (Promega) and ECL® (enhanced chemiluminescence reagent; GE Healthcare). For quantification of signals, the images were scanned, imported into an Odyssey imaging system (LI-COR Biosciences) and quantified.

### Glucose tolerance test, pyruvate tolerance test and insulin tolerance test

After mice were deprived of food for 4 h (before the insulin tolerance test) or overnight (16 h; for glucose and pyruvate tolerance tests), basal blood glucose levels were measured via tail bleeding using a Breeze 2 glucometer (Bayer). For glucose and pyruvate tolerance tests, the mice were intraperitoneally injected with a bolus of glucose or pyruvate (2 mg of glucose or pyruvate per g of body weight). For insulin tolerance tests, the mice were subject to a bolus injection of insulin (0.75 m-units of insulin/g of body weight). Blood glucose levels were subsequently measured at the times indicated.

### Hyperinsulinaemic–euglycaemic clamp

All procedures required for the hyperinsulinaemic–euglycaemic clamp were approved by the Vanderbilt University Animal Care and Use Committee. Catheters were implanted into a carotid artery and a jugular vein of mice for sampling and infusions respectively 5 days before the study [24]. Insulin clamps were performed on mice fasted for 5 h. [3-^3^H]Glucose was primed (2.4 μCi) and continuously infused for a 90-min equilibration period (0.04 μCi/min) and a 2-h clamp period (0.12 μCi/min). Baseline blood or plasma parameters were determined as the mean of values obtained in blood samples collected at −15 and −5 min. At time 0, insulin infusion (2.5 m-units/kg of body weight per min) was started and continued for 165 min. Blood glucose was clamped using a variable rate of glucose infusion, which was adjusted based on the measurement of blood glucose at 10 min intervals. Mice received heparinized saline-washed erythrocytes from donors at 5 μl/min to prevent a fall in haematocrit. Insulin clamps were validated by assessment of blood glucose over time. Blood was taken at 80–120 min for the determination of [3-^3^H]glucose. Clamp insulin was determined at *t*=100 and 120 min. At 120 min, 13 μCi of 2-[^14^C]deoxyglucose (abbreviated to [^14^C]2DG) was administered as an intravenous bolus. Blood was taken at 2, 15, 25 and 35 min after the bolus for the determination of [^14^C]2DG. After the last sample, mice were anaesthetized and tissues were collected.

### Insulin clamp plasma and tissue sample processing

Plasma insulin was determined by ELISA (Millipore). Radioactivity of [3-^3^H]glucose, [^14^C]2DG and [^14^C]2DG-6-phosphate in plasma and tissue samples were determined by liquid-scintillation counting [25]. Glucose appearance and disappearance rates were determined using non-steady-state equations [26]. Endogenous glucose production was determined by subtracting the GIR (glucose infusion rate) from the total glucose production. The glucose metabolic index was calculated as described previously [27].

### Indirect calorimetry measurement

Male mice at 8 weeks of age were individually housed in and adapted to metabolic cages (Columbus Instruments) for 24 h before indirect calorimetry measurements. Oxygen consumption (*V̇*O_2_), carbon dioxide production (*V̇*CO_2_), physical activity (*X*_AMB_) and food intake were monitored using Oxymax/CLAMS (Columbus Instruments). Data were collected for 2 days and averaged for these 2 days. The RER (respiratory exchange ratio) is calculated as:

$$\mathrm{RER}=\dot{V}{\mathrm{co}}_{2}/\dot{V}{\mathrm{co}}_{2}$$

Heat production (kcal/unit time; 1 kcal=4.184 kJ) was calculated using the following equation:

$$\mathrm{Heat}=(3.815+1.232\times \mathrm{RER})\times \dot{V}o_{2}$$

### Muscle incubation and glucose uptake *ex vivo*

Soleus or EDL (extensor digitorum longus) muscles were isolated and incubated with or without insulin in KRB (Krebs–Ringer bicarbonate) buffer for 50 min as described previously [12]. After incubation, the muscles were either snap-frozen in liquid nitrogen to study signalling proteins or used for the glucose-uptake assay as described previously [28]. After glucose uptake, the muscles were snap-frozen, weighed and processed as described previously [28]. Radioisotopes in the muscles were counted using a Tri-Carb 2800TR scintillation counter (PerkinElmer).

### Glucose uptake in primary adipocytes

Primary adipocytes were isolated from epididymal fat pads as described previously [12]. Cells were incubated with or without insulin at 37°C for 30 min. Glucose uptake was subsequently carried out at 37°C for 30 min in the presence or absence of insulin, and stopped by adding cytochalasin B as described previously [12]. Cell suspension was centrifuged through dinonylphtalate oil and used for scintillation counting with a Tri-Carb 2800TR scintillation counter (PerkinElmer).

### Statistical analysis

Data analysis was carried out using Student's *t* test, and differences were considered statistically significant at *P*<0.05.

## RESULTS

### Generation of an AS160-knockout mouse

**Figure 1:**
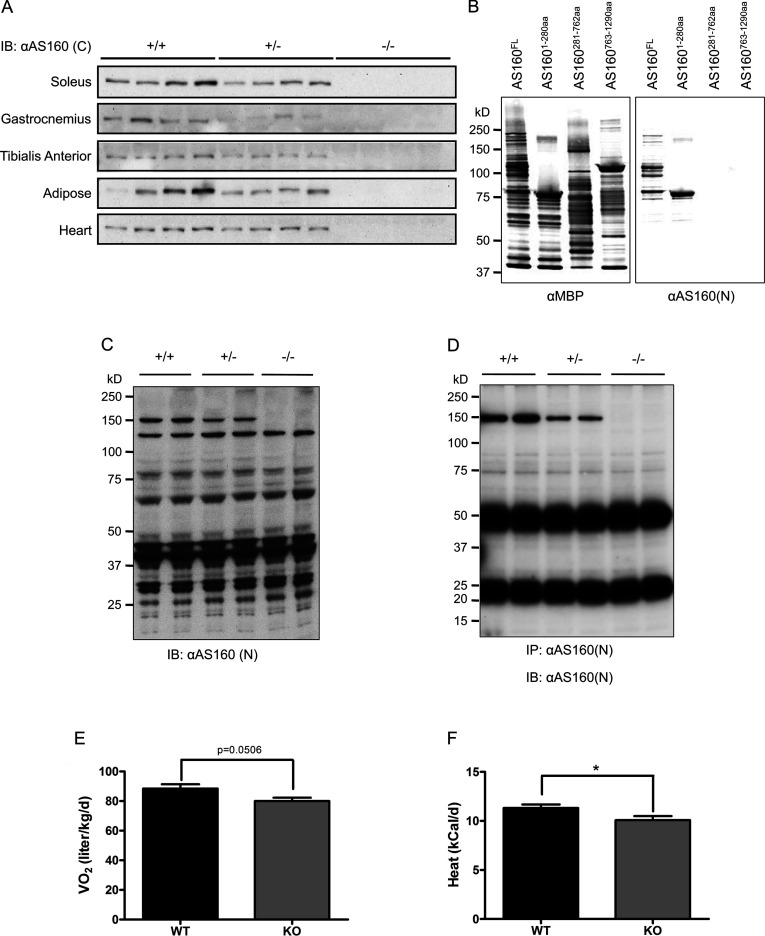
Generation and basic characterization of the AS160-knockout mouse (**A**) AS160 expression in various tissues. Various tissues were removed from 8-week-old male mice. Full-length AS160 proteins were measured in 40 μg of tissue lysates using the commercial anti-AS160(C) antibody that was raised against a C-terminal peptide of AS160. (**B**) Characterization of the anti-AS160(N) antibody. MBP-tagged human AS160 proteins (full-length and fragments) were expressed in and purified from *Escherichia coli*, and subjected to Western blotting analysis (300 ng/lane). In the left-hand panel, the anti-MBP antibody was used to detect the MBP-tagged recombinant proteins. In the right-hand panel, the anti-AS160 (N) antibody was used for detection. (**C**) Expression of full-length AS160 and truncated AS160^1–609^ in soleus muscle. Soleus muscle was removed from 8-week-old male mice. Full-length AS160 proteins and truncated AS160^1–609^ fragment were measured in 40 μg of soleus lysates using the anti-AS160(N) antibody that is raised agaist the N-terminus (residues 1–280) of human AS160. (**D**) Immunoprecipitation of full-length AS160 and truncated AS160^1–609^ from tissue lysates of gastrocnemius (GAS) muscle. GAS muscle was removed from 8-week-old male mice. Full-length AS160 proteins and truncated AS160^1–609^ fragment (if the latter had been present) were immunoprecipitated from 500 μg of GAS lysates using the anti-AS160(N) antibody. The immunoprecipitates were analysed using Western blotting with the anti-AS160(N) antibody (used at 1 μg/ml at 4°C overnight). (**E**) Oxygen consumption during a metabolic cage study. The AS160-knockout and wild-type male mice (8 weeks old) were monitored using an Oxymax/CLAMS system (Columbus Instruments). The data are given as the means±S.E.M. (*n*=6–7). (**F**) Heat production during a metabolic cage study. The AS160-knockout and wild-type male mice (8 weeks old) were monitored using an Oxymax/CLAMS system (Columbus Instruments). The data are given as the means±S.E.M. (*n*=6–7). * *P*<0.05. For (**B**–**D**) the molecular mass in kDa is indicated on the left-hand side. IB, immunoblot; IP, immunoprecipitation; KO, knockout; WT, wild-type.

We previously reported the generation of an AS160^T649A^ knockin mouse in which the tenth exon harbouring a T649A mutation is flanked by loxP-Cre excision sites [12]. This strategy allows AS160-knockout mice to be generated by mating the AS160^T649A^ knockin mouse with the Bal1 mouse line that expresses Cre recombinase in all tissues [21]. The tenth exon of the AS160 T649A allele was excised in the offspring of such matings (depicted in Supplementary Figure S1A at http://www.biochemj.org/bj/449/bj4490479add.htm), resulting in a shift in the open reading frame of AS160. Theoretically, this frame-shift could produce a peptide spanning amino acids 1–609 of AS160 plus a short sequence of HCPVSPAHVQGE in the AS160-knockout mouse. The expression of full-length AS160 was decreased in the tissues from the heterozygous AS160-knockout mouse, and undetectable in the tissues from the homozygous AS160-knockout mouse using a commercial AS160 antibody raised against a C-terminal peptide of AS160, here termed anti-AS160(C) (Figure 1A). To search for expression of the theoretical truncated AS160^1–609^, we also generated an antibody that specifically recognized the N-terminus of AS160 (amino acids 1–280), termed anti-AS160(N) (Figure 1B). Using this antibody, we failed to find any AS160^1–609^ fragment in extracts of soleus muscle from the AS160-knockout mouse (Figure 1C). Moreover, the anti-AS160(N) antibody could immunoprecipitate the full-length AS160 protein from various tissues of the wild-type mouse, less full-length protein and no detectable AS160^1–609^ fragment from various tissues of the heterozygous AS160-knockout mouse, and more importantly no detectable full-length and AS160^1–609^ fragment from various tissues of the homozygous AS160-knockout mouse (Figure 1D and Supplementary Figures 1B–1D). These data showed that the AS160-knockout mouse generated in the present study was suitable for addressing the effects of loss-of-function of AS160 on glucose metabolism.

### Basic characterization of the AS160-knockout mouse

The AS160-knockout mouse was viable and exhibited no gross physical abnormalities. Body weight gain of the AS160-knockout mouse was normal within the monitoring period (Supplementary Figure S2 at http://www.biochemj.org/bj/449/bj4490479add.htm). However, blood glucose levels under both randomly fed and fasted conditions were lower, whereas plasma non-esterified (free) fatty acid levels were slightly higher under fasted conditions in the AS160-knockout mice compared with the wild-type littermates (Table 1). There was no change in the plasma triacylglycerol levels under randomly fed conditions, and muscle glycogen levels were also normal in the AS160-knockout mouse (Table 1). The plasma insulin level was normal under fasted conditions, and slightly lower under randomly fed conditions in the AS160-knockout mouse (Table 1). The plasma adiponectin level was slightly lower under fasted conditions, and significantly lower under randomly fed conditions in the AS160-knockout mouse (Table 1). The AS160-knockout mouse had normal food intake and physical activities (Supplementary Figures S1G and S1H), but a decreased rate of energy expenditure as shown by lower rates of oxygen consumption and heat production (Figures 1E and 1F).

**Table 1:** Basic characterization of the AS160-knockout mouse Plasma insulin, blood glucose, adiponectin and NEFA [non-esterified (‘free’) fatty acid] concentrations were measured in 10–12-week-old male mice after fasting (5 h in the case of NEFAs, and 16 h for plasma insulin and adiponectin, and blood glucose measurements) and random-fed conditions. Glycogen levels were determined in TA muscle from 6-week-old mice after partial fasting (5 h). Values are given as means±S.E.M. from at least six animals. n.d., not determined. *P<0.05 and §*P*=0.094.

	Fasted		Fed	
Measurement	Wild-type	Knockout	Wild-type	Knockout
Insulin (ng/ml)	0.226±0.010	0.232±0.019	0.930±0.169	0.728±0.037
Blood glucose (mM)	7.3±0.4	5.9±0.3*	9.8±0.2	9.0±0.4
Adiponectin (ng/ml)	10.0±0.8	8.5±0.4§	11.4±0.3	9.2±0.4*
NEFA (mM)	1.062±0.066	1.220±0.106	0.898±0.062	0.938±0.086
Triacylglycerol (mM)	n.d.	n.d.	0.704±0.037	0.709±0.065
Muscle glycogen (μmol/g)	15.8±2.6	16.6±2.8	n.d.	n.d.

### Insulin signalling in muscle and adipose tissue is normal in the AS160-knockout mouse

**Figure 2:**
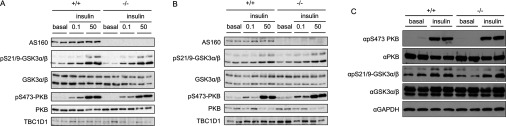
Insulin-stimulated phosphorylation of GSK3 and PKB in muscle and adipose tissue Soleus (**A**) and EDL (**B**) muscles isolated from 8-week-old mice were incubated in the absence or presence of insulin (0.1 or 50 m-units/ml) for 50 min. Adipose tissue (**C**) was collected from 8-week-old mice that were intraperitoneally injected with 150 m-units/g insulin or saline for 20 min. Full-length AS160 was detected in 40 μg of muscle lysates using the commercial anti-AS160 antibody that recognizes the C-terminus of AS160. The phosphorylation of GSK3 and PKB, and total GSK3, PKB and TBC1D1, and GAPDH were determined in 40 μg of muscle or adipose lysates by using the respective phospho-specific antibodies or total antibodies indicated in the Materials and methods section.

Insulin signalling was analysed in isolated muscles from the AS160-knockout mice and wild-type littermates using two doses of insulin (submaximal dose, 0.1 m-units/ml; maximal dose, 50 m-units/ml). Phosphorylation of PKB and its substrate GSK3α/β was normal in soleus muscle from the AS160-knockout mouse with both doses of insulin (Figure 2A), suggesting that proximal insulin signalling was intact in the AS160-knockout mouse. Moreover, the expression level of the related RabGAP TBC1D1 was not altered in soleus muscle from the AS160-knockout mouse (Figure 2A). Similar results were obtained in isolated EDL muscle (Figure 2B). Insulin-stimulated phosphorylation of PKB and GSK3α/β was also normal in adipose tissue from the AS160-knockout mouse (Figure 2C). Taken together, these data suggest that the AS160-knockout mouse described in the present study is a useful model to study the effect of AS160 deletion on glucose metabolism.

### The AS160-knockout mouse is glucose-tolerant but exhibits reduced insulin sensitivity

**Figure 3:**
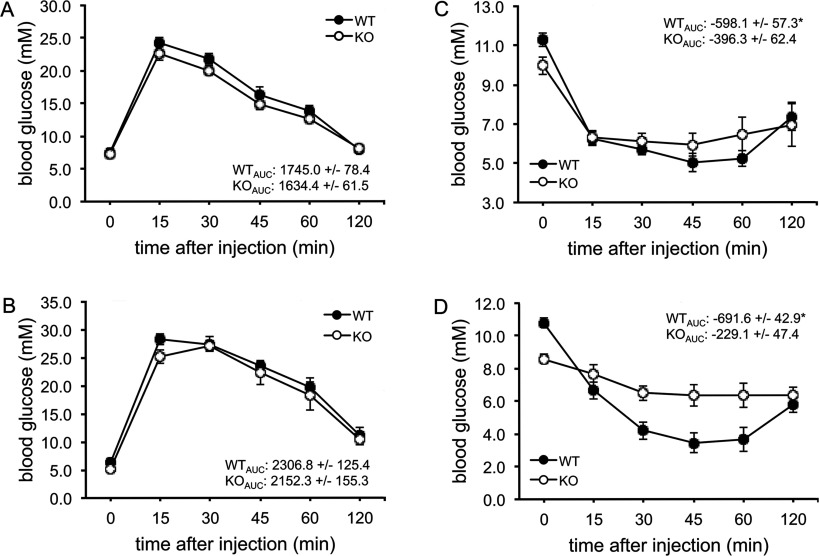
Glucose clearance in the AS160-knockout mouse after intraperitoneal injection with glucose or insulin (**A**) Glucose tolerance test of male mice at 8 weeks of age. The values show the glucose area under the curve during the glucose tolerance test. The data are given as the means±S.E.M. (*n*=5–6). (**B**) Glucose tolerance test of male mice at 12 weeks of age. The values show the glucose area under the curve during the glucose tolerance test. The data are given as the means±S.E.M. (*n*=7). (**C**) Insulin tolerance test of male mice at 7–8 weeks of age. The values show the glucose area above the curve during the insulin tolerance test. The data are given as the means±S.E.M. (*n*=6–7). *P<0.05. (**D**) Insulin tolerance test of male mice at 16–17 weeks of age. The values show the glucose area above the curve during the insulin tolerance test. The data are given as the means±S.E.M. (*n*=8). *P<0.05. AUC, area under the curve; KO, knockout; WT, wild-type.

We next investigated the effects of knocking out AS160 on whole-body glucose homoeostasis. The AS160-knockout mice and wild-type littermates were fasted overnight (16 h) and injected intraperitoneally with a bolus of glucose (2 mg of glucose/g of body weight). The blood glucose levels reached similar peaks and then declined at similar rates in the two genotypes at ages of 2 and 3 months (Figures 3A and 3B). Plasma insulin levels after glucose injection were normal in the AS160-knockout mouse (Supplementary Figure S3 at http://www.biochemj.org/bj/449/bj4490479add.htm). The AS160-knockout mice and wild-type littermates were partially fasted for 4 h and then intraperitoneally injected with a bolus of insulin (0.75 m-units/g of body weight). Consistent with the aforementioned data (Table 1), the blood glucose level was significantly lower in the AS160-knockout mice than in wild-type littermates after their 4 h fast. Interestingly, insulin did not decrease blood glucose levels in the AS160-knockout mice as efficiently as in the wild-type littermate controls at 2 and 4 months (Figures 3C and 3D), suggesting that the AS160-knockout mice are insulin resistant.

**Figure 4:**
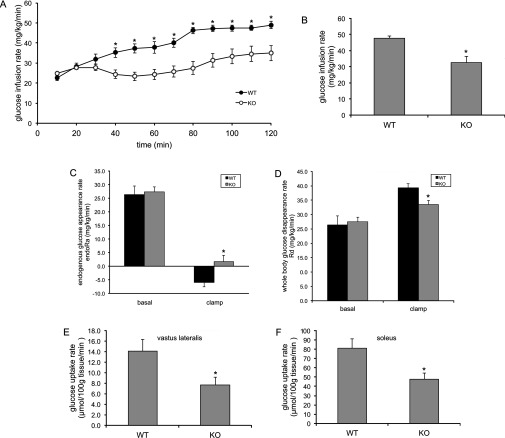
GIR, endogenous glucose production (endoR_a_), whole-body glucose disappearance (R_d_) and tissue glucose uptake during hyperinsulinaemic–euglycaemic clamp (**A**) GIR during euglycaemic clamp in the AS160-knockout and wild-type male mice (16 weeks old). Data are given as means±S.E.M. for six (wild-type) or ten (knockout) mice. *P<0.05. (**B**) Steady-state GIR obtained from averaged rates of 80–120 min of hyperinsulinaemic–euglycaemic clamps in the AS160-knockout mice and wild-type littermates. Data are given as means±S.E.M. for six (wild-type) or ten (knockout) mice. *P<0.05. (**C**) Endogenous glucose appearance rates (endoR_a_) during basal conditions and euglycaemic clamps. Basal endoR_a_ is obtained from averaged values at *t*=−10 and 0 min before onset of insulin infusion. Clamp endoR_a_ is obtained from averaged rates of 80–120 min of euglycaemic clamps. Data are given as means±S.E.M. for six (wild-type) or ten (knockout) mice. *P<0.05. (**D**) Whole-body glucose disappearance rates (R_d_) during basal conditions and euglycaemic clamps. Basal R_d_ is obtained from averaged values at t=−10 and 0 min before onset of insulin infusion. Clamp R_d_ is obtained from averaged rates of 80–120 min of euglycaemic clamps. Data are given as means±S.E.M. for six (wild-type) or ten (knockout) mice. *P<0.05. (**E** and **F**) Insulin-stimulated glucose uptake during euglycaemic clamps in skeletal muscle [vastus lateralis (**E**) and soleus (**F**)] from the AS160-knockout mouse and wild-type littermates. Data are given as means±S.E.M. for six (wild-type) or ten (knockout) mice. *P<0.05. KO, knockout; WT, wild-type.

To identify the site(s) of insulin resistance in the AS160-knockout mouse, we carried out a hyperinsulinaemic–euglycaemic clamp. In response to constant insulin infusion, glucose was infused at a variable rate to maintain blood glucose levels that were tightly controlled at similar levels (~5.5 mM) in the AS160-knockout and wild-type mice (Supplementary Figure S4A at http://www.biochemj.org/bj/449/bj4490479add.htm). Plasma insulin levels were comparable in the AS160-knockout and wild-type mice before the clamp, and were significantly elevated to a similar extent in the two genotypes during the clamp (Supplemenatry Figure S4B). The glucose infusion rates required to maintain euglycaemia were significantly lower in the AS160-knockout mice than the wild-type littermates (Figures 4A and 4B), again suggesting that the AS160-knockout mice were insulin-resistant, consistent with the reduced insulin sensitivity revealed by the insulin tolerance test. The rates of endogenous glucose production were comparable in the two genotypes before the clamp; however, they were significantly higher in the AS160-knockout mice than the wild-type littermates during the clamp (Figure 4C), suggesting that insulin-induced suppression of hepatic glucose production was impaired in the AS160-knockout mice. The rates of glucose disposal were normal in the AS160-knockout mice before the clamp, but significantly lower in the AS160-knockout mice compared with wild-type littermates during the clamp (Figure 4D). Consistent with skeletal muscle accounting for the majority of glucose disposal under such conditions, the glucose uptake rates during the clamp were significantly lower in skeletal muscle of the AS160-knockout mice than the wild-type littermates (Figure 4E and 4F). Taken together, impaired muscle and liver insulin action accounts for the reduced whole-body insulin sensitivity in the AS160-knockout mouse.

### The AS160-knockout mouse has altered hepatic function and gluconeogenetic enzyme expression

**Figure 5:**
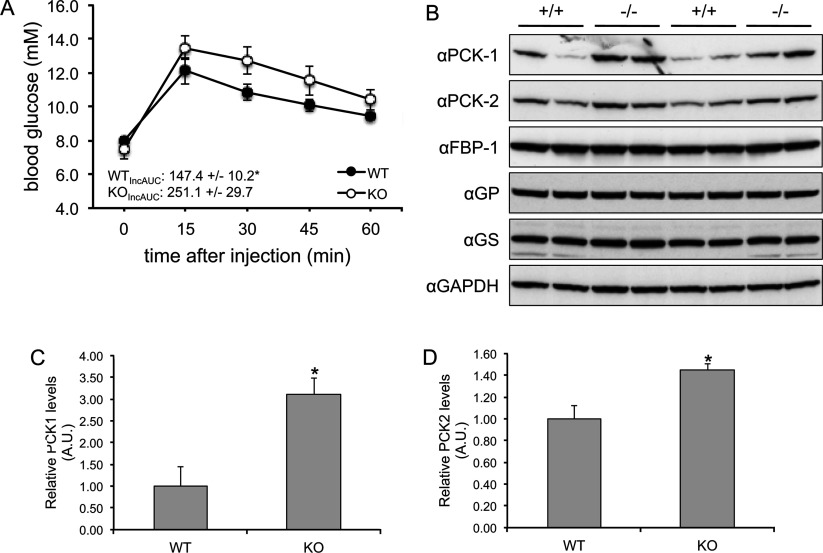
Pyruvate tolerance test and hepatic PEPCK expression (**A**) Pyruvate tolerance test of the AS160-knockout and wild-type male mice at 9–10 weeks of age. After deprivation of food for 16 h (overnight), the mice were intraperitoneally injected with a bolus of pyruvate (2 mg/g of body weight). At zero time, and at the indicated times after injection, blood glucose levels were determined with a Breeze 2 glucometer (Bayer). The incremental area under the curve (IncAUC) was calculated. Data are given as the means±S.E.M. (*n*=4 for each genotype). *P<0.05. (**B**–**D**) PEPCK protein expression in liver from the AS160-knockout and wild-type mice. Liver lysates (40 μg) were separated by SDS/PAGE and subject to Western blot analysis. The levels of cytosolic (anti-PCK-1) and mitochondrial (anti-PCK-2) isoforms of PEPCK, FBP-1, glycogen synthase (GS), glycogen phosphorylase (GP) and GAPDH were measured using the antibodies indicated. The PEPCK signals were quantified and normalized with GAPDH. Data are given as means±S.E.M. (*n*=4). *P<0.05. A.U., arbitrary unit; KO, knockout; WT, wild-type.

To investigate the mechanisms underlying the hepatic insulin resistance in the AS160-knockout mouse, we carried out a pyruvate tolerance test in which the mice were injected intraperitoneally with a bolus of pyruvate (2 mg of pyruvate/g of body weight) after deprivation of food for 16 h (overnight). The AS160-knockout mice were intolerant to pyruvate challenge (Figure 5A), indicating they might have increased hepatic gluconeogenesis. In line with this, the expression of both cytosolic and mitochondrial isoforms of PEPCK, a key enzyme in gluconeogenesis, were significantly increased in liver from the AS160-knockout mice (Figures 5B–5D).

### Tissue-dependent impairment of glucose uptake in primary adipocytes and isolated skeletal muscle from the AS160-knockout mouse

**Figure 6:**
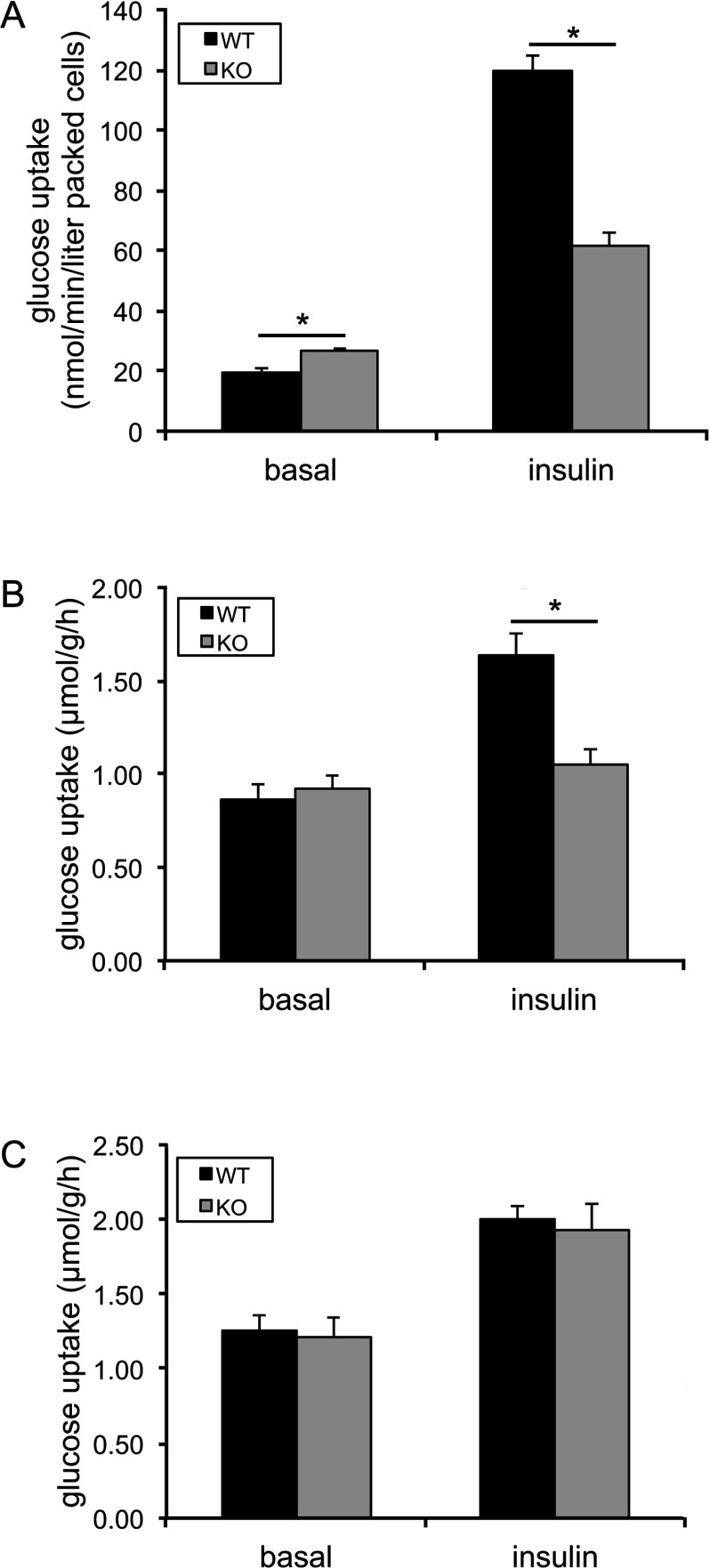
Insulin-stimulated glucose uptake in primary adipocytes and muscle *ex vivo* (**A**) Insulin-stimulated glucose uptake in primary adipocytes. The primary adipocytes isolated from epididymal fat pads of male mice (16 weeks old) were stimulated with or without 16.7 m-units/ml insulin for 30 min and used for the glucose uptake assay. Data are given as means±S.E.M. (*n*=9–10). *P<0.05. (**B**) Insulin-stimulated glucose uptake in soleus muscle *ex vivo*. Soleus muscle isolated from the AS160-knockout and wild-type mice (10–16 weeks old) was stimulated with or without 0.1 m-units/ml insulin and used for the glucose uptake assay. Data are given as means±S.E.M. (*n*=13–14). *P<0.05. (**C**) Insulin-stimulated glucose uptake in EDL muscle *ex vivo*. EDL muscle isolated from the AS160-knockout and wild-type mice (6–11 weeks old) was stimulated with or without 0.5 m-units/ml insulin and used for the glucose uptake assay. Data are given as means±S.E.M. (*n*=5–7). KO, knockout; WT, wild-type.

To elucidate the mechanisms underlying the diminished glucose disposal and decreased insulin sensitivity in the AS160-knockout mice, we next measured the rate of glucose uptake into primary adipocytes and isolated skeletal muscle. In primary adipocytes isolated from epididymal fat pad, glucose uptake under basal conditions was significantly increased by nearly 40%, whereas insulin-stimulated glucose uptake was decreased by 50% in AS160-knockout cells compared with wild-type cells (Figure 6A). In isolated soleus muscle, glucose uptake under unstimulated conditions was comparable in the AS160-knockout and wild-type littermates (Figure 6B). Insulin stimulated glucose uptake by nearly 2-fold in isolated soleus muscle from the wild-type mice, whereas the insulin response was almost completely blunted in soleus muscle from the AS160-knockout mice (Figure 6B). In isolated EDL muscle, glucose uptake under unstimulated conditions was also comparable in the two genotypes (Figure 6C). In contrast with isolated soleus muscle, glucose uptake was stimulated by insulin to a similar extent (~60%) in isolated EDL muscles from the wild-type and AS160-knockout mice (Figure 6C).

### Tissue-dependent impairment of GLUT4 expression in adipose tissue and skeletal muscle from the AS160-knockout mouse

**Figure 7:**
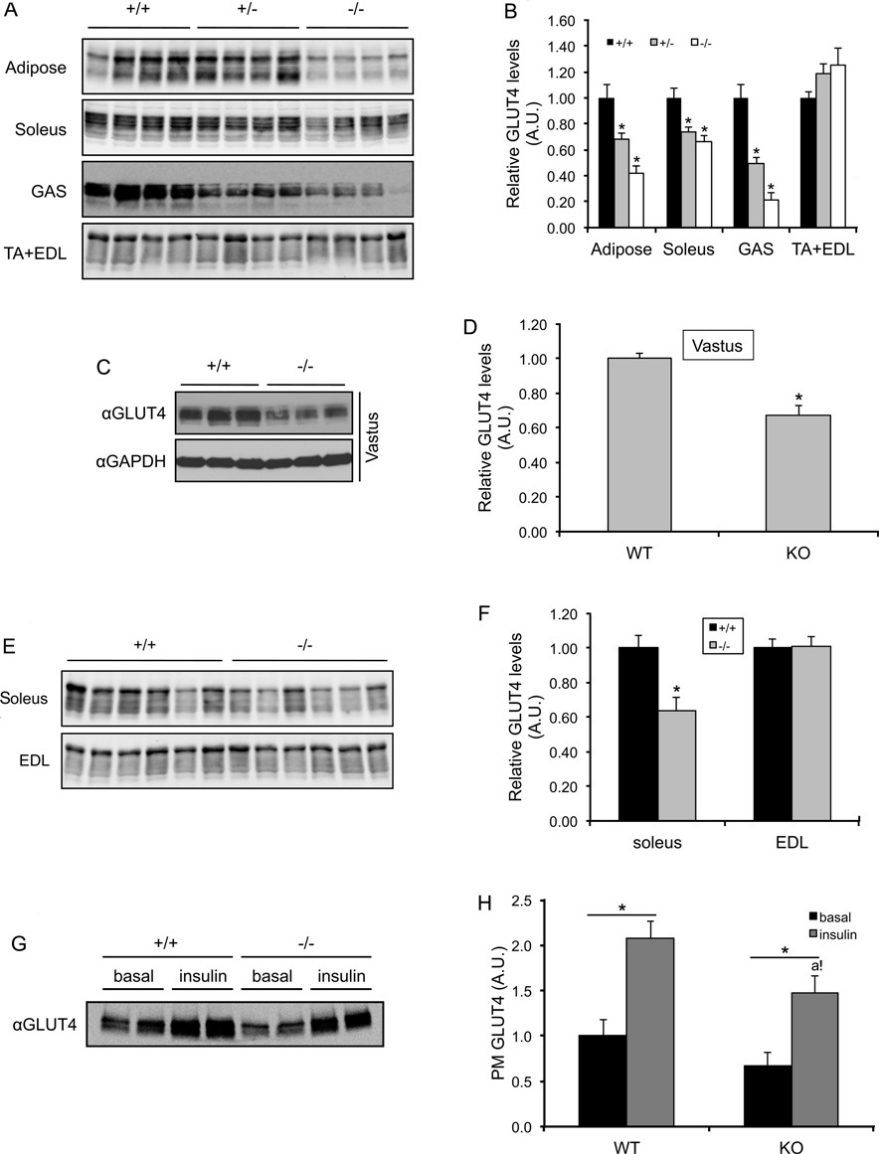
Total and PM-associated GLUT4 levels in adipose tissue and skeletal muscle (**A**–**F**) Total GLUT4 levels in adipose tissue and various skeletal muscles from the AS160-knockout and wild-type mice (8 weeks old). GLUT4 proteins were detected in 40 μg of total lysates of adipose tissue, and skeletal muscle using an anti-GLUT4 antibody (**A**, **C** and **E**) and the signals were quantified (**B**, **D** and **F**). Data are given as means±S.E.M. (**B**, *n*=4; **D**, *n*=7–9; **F**, *n*=6). *P<0.05. (**G**–**H**) GLUT4 levels on the PM of adipose tissue. Adipose tissue was collected from 8-week-old mice that were intraperitoneally injected with 150 m-units/g insulin or saline (basal) for 20 min. Subcellular fractionation of adipose tissue was carried out as described in Materials and methods section. The GLUT4 levels in the PM fraction was determined via Western blot analysis using an anti-GLUT4 antibody (**G**) and the signals were quantified (**H**). Data are given as means±S.E.M. (*n*=3–4). *P<0.05; a! indicates *P*<0.05 compared with WT (insulin). A.U., arbitrary unit; GAS, gastrocnemius; KO, knockout; WT, wild-type.

To gain insights into the changes in glucose uptake in primary adipocytes and skeletal muscle, we determined the expression levels of GLUT4 in adipose tissue and skeletal muscle, which is the major mediator of insulin-stimulated glucose uptake in these tissues. Interestingly, the GLUT4 levels were significantly decreased in adipose tissue (epididymal fat), soleus, gastrocnemius and vastus muscle from the AS160-knockout mouse compared with the wild-type littermates, whereas they were comparable in EDL and TA (tibialis anterior) muscle from the two genotypes (Figures 7A–7F). The GLUT4 levels correlated with the selective impairment of glucose uptake in primary adipocytes, and soleus, vastus and EDL muscle. In contrast, GLUT1 levels were normal in both adipose tissue and skeletal muscle (Supplementary Figure S5 at http://www.biochemj.org/bj/449/bj4490479add.htm).

Since AS160 plays a key role in regulating GLUT4 trafficking, we next performed subcellular fractionation of adipose tissue to investigate the effect of AS160 deficiency on insulin-stimulated GLUT4 translocation. The GLUT4 levels on the PM were lower in adipose tissue from the AS160-knockout mice at both basal and insulin-stimulated states (Figures 7G and 7H), whereas its fold-increase upon insulin stimulation was similar in the two genotypes (~2-fold in both genotypes; Figures 7G and 7H), suggesting that AS160 deficiency does not impair insulin-stimulated GLUT4 translocation itself, and the lower GLUT4 levels on the PM and glucose uptake upon insulin stimulation were most likely due to the decreased expression levels of GLUT4 in the AS160-knockout mice.

## DISCUSSION

In the present study, we generated an AS160-knockout mouse model to study the effects of AS160 deletion on glucose homoeostasis in mouse, which reveals similarities and differences between the AS160-knockout mouse and the human patients bearing the AS160 truncation mutation [19]. For example, both the AS160-knockout mouse and humans with the AS160 truncation developed insulin resistance. Unlike the human patient who displays postprandial hyperinsulinaemia, the AS160-knockout mouse did not develop postprandial hyperinsulinaemia, but had rather normal plasma insulin levels. Detailed investigation of the AS160-knockout mouse revealed the mechanisms underlying the insulin resistance caused by AS160 deletion. Measurement of whole-body glucose fluxes and analyses of skeletal muscle and adipose tissue uncovered new aspects of AS160 in regulation of glucose homoeostasis in these tissues.

Experimentally, a peptide (amino acids 1–362) of AS160, corresponding to the truncated protein that might be expressed in the human patients with the AS160-R363X mutation, was able to inhibit insulin-stimulated GLUT4 translocation when overexpressed in 3T3-L1 adipocytes [19]. Recently, it has also been shown that a longer peptide (amino acids 1–532) of AS160 could inhibit insulin-stimulated GLUT4 vesicle fusion with the plasma membrane *in vitro* [29]. In both cases, the inhibitory effects of these two peptides of AS160 on GLUT4 translocation or GLUT4 vesicle fusion were proposed to be mediated through dimerization with the full-length AS160 and therefore interference with its function in a dominant-negative manner [19,29]. In the latter case, the peptide dimerized with the full-length AS160 and consequently disrupted its interaction with 14-3-3 protein [29], which plays an important role in insulin-stimulated GLUT4 trafficking [12]. However, these mechanisms cannot explain the lower insulin-stimulated glucose uptake in primary adipocytes and certain types of skeletal muscle from the AS160-knockout mouse (Figures 4E, 4F, 6A and 6B) because the theoretical truncated peptide of AS160 was not detectable and there was no full-length AS160 in the homozygous AS160-knockout mouse (Figures 1B–1D).

We found that the GLUT4 levels were decreased in adipose tissue, and soleus, gastrocnemius and vastus muscle, whereas they remained normal in EDL and TA muscle in the AS160-knockout mice (Figures 7A–7F). This tissue-dependent effect on GLUT4 levels in adipose tissue and skeletal muscle correlates with the changes in insulin-stimulated glucose uptake in primary adipocytes and various types of skeletal muscle (Figures 4E–4F and 6A–6C), suggesting that the decreased glucose uptake in response to insulin in primary adipocytes, and soleus and vastus muscle from the AS160-knockout mouse is most probably caused by the lower levels of GLUT4 in these cells and tissues. During the revision of the present paper, another AS160-knockout mouse model was reported, in which GLUT4 levels were also decreased in a similar tissue-dependent fashion [30]. But why does the deletion of AS160 affect the GLUT4 levels in a tissue-dependent manner? We do not know the answer yet, although we have noticed that the tissue-dependent effect of AS160 deletion on GLUT4 levels correlates with the expression levels of AS160 in wild-type mice. The expression levels of AS160 are higher in adipose tissue, and soleus and vastus muscle than that in TA and EDL muscle (Supplementary Figure S6 at http://www.biochemj.org/bj/449/bj4490479add.htm). The effect of AS160 deletion on GLUT4 levels is reminiscent of the effect of a related RabGAP, TBC1D1, whose deletion in mouse also results in a decrease in GLUT4 levels in skeletal muscle [31]. In contrast, GLUT4 levels were elevated in muscle and adipose tissue in the AS160^T649A^ knockin mouse [12]. These observations raise two questions to be addressed in the future. First, how does manipulation of these two related RabGAPs, namely AS160 and TBC1D1, affect the GLUT4 levels? Another critical question is whether deletion of AS160 and TBC1D1 affect the same or different pools of GLUT4.

The decreased GLUT4 levels also affected the *in vivo* glucose uptake in skeletal muscle (Figures 4E and 4F) which, together with impaired insulin-induced suppression of hepatic glucose production, contributed to the decreased insulin sensitivity in the AS160-knockout mouse (Figures 2C, 2D and 4A). However, one puzzle is that the insulin resistance in skeletal muscle and liver did not result in glucose intolerance and hyperinsulinaemia in the AS160-knockout mouse. The AS160-knockout mouse displayed normal plasma insulin levels after intraperitoneal injection of glucose (Supplementary Figure S3), suggesting that there was no compensation from plasma insulin levels to regulate glucose homoeostasis in the AS160-knockout mouse after glucose injection. Another conundrum is that blood glucose levels under both randomly fed and fasting conditions were lower, despite the insulin resistance and decreased GLUT4 levels, in the AS160-knockout mouse. These two puzzles suggest that there must be a mechanism only operating under conditions (such as feeding and glucose injection) that elevate plasma insulin levels within physiological ranges via insulin secretion from pancreatic β-cells, but not under conditions (such as insulin tolerance test and insulin clamp) that result in hyperinsulinaemia independent of insulin secretion from pancreatic β-cells. Nevertheless, these metabolic changes in the AS160-knockout mouse are reminiscent of the phenotype of the muscle-specific insulin-receptor-knockout mouse that is glucose tolerant and maintains euglycaemia, but displays insulin resistance in euglyacemic clamp studies [32,33].

While AS160 is expressed in most tissues analysed, the protein was undetectable in liver extracts (Supplementary Figure S6), although an AS160-derived peptide was identified in a digest of 14-3-3 affinity-purified proteins from liver (M. Tinti and C. MacKintosh, unpublished work). Nevertheless, liver in the AS160-knockout mouse developed insulin resistance (Figure 4B), suggesting that either there is an important ‘trace’ expression of AS160 in liver, or perhaps, more likely, there is tissue ‘cross-talk’ in the AS160-knockout mouse. Insulin resistance caused by tissue ‘cross-talk’ is commonly found in GLUT4-knockout mouse models. For instance, in the adipose-specific GLUT4-knockout mouse, liver and skeletal muscle also develop insulin resistance that was speculated to be caused by an unidentified adipocyte-derived molecule [34,35]. In the muscle-specific GLUT4-knockout mouse, insulin actions in adipose tissue and liver are also impaired, which is at least partly due to glucose toxicity [35–37]. In contrast, overexpression of GLUT4 in muscle and fat causes increased hepatic insulin actions in the fasted transgenic mice [38]. Since the GLUT4 levels were decreased in certain types of muscle and adipose tissue in the AS160-knockout mouse, one possibility is that adipose tissue with the decreased GLUT4 levels might cross-talk with the liver and result in hepatic insulin resistance in the AS160-knockout mouse. Another possibility is that AS160 in certain tissue(s) might modulate a messenger that can cross-talk with the liver and regulate hepatic insulin sensitivity, and deletion of AS160 might disrupt such cross-talk. In support of this notion, the levels of adiponectin, which is a known factor modulating insulin sensitivity [39], were lower in the plasma of the AS160-knockout mouse (Table 1), although the importance of adiponectin in the development of hepatic insulin resistance in the AS160-knockout mouse is still to be investigated.

In summary, deletion of AS160 has an impact on glucose homoeostasis in mouse by: (i) decreasing GLUT4 levels in certain types of skeletal muscle and adipose tissue, thus resulting in a lower glucose uptake rate and (ii) causing hepatic insulin resistance through an unknown inter-organ communication process. Consequently, these effects render the AS160-knockout mouse less sensitive to insulin.

## Online data

Supplementary data
