# Supplementary material for: AS160 deficiency causes whole-body insulin resistance via composite effects in multiple tissues
Source: Biochem J. 2012 Dec 14;449(Pt 2):479–89. doi: 10.1042/BJ20120702 (PMC3685216; doi:10.1042/BJ20120702)
Supplement: Supplementary data [file bj4490479add.pdf]

BJ Metabolism

Biochem. J. (2013) **449**, 479–489 (Printed in Great Britain) doi:10.1042/BJ20120702

online data

SUPPLEMENTARY ONLINE DATA

# AS160 deficiency causes whole-body insulin resistance via composite effects in multiple tissues

Hong Yu WANG*, Serge DUCOMMUN†‡, Chao QUAN*, Bingxian XIE*, Min LI*, David H. WASSERMAN§, Kei SAKAMOTO†‡, Carol MACKINTOSH†‖[1] and Shuai CHEN*[1]

*MOE Key Laboratory of Model Animal for Disease Study, Model Animal Research Center, Nanjing University, Pukou District, Nanjing 210061, China, †MRC Protein Phosphorylation Unit, College of Life Sciences, University of Dundee, Dundee DD1 5EH, Scotland, U.K., ‡Nestlé Institute of Health Sciences SA, Campus EPFL, Quartier de l'Innovation, Bâtiment G, 1015 Lausanne, Switzerland, §Department of Molecular Physiology and Biophysics, Vanderbilt University, School of Medicine, 2200 Pierce Ave, Nashville, TN 37232, U.S.A., and ‖Division of Cell and Developmental Biology, College of Life Sciences, University of Dundee, Dundee DD1 5EH, Scotland, U.K.

Supplementary Figures S1–S6 are on the following pages

Biochemical Journal www.biochemj.org

[1] Correspondence may be addressed to either of the senior authors (email c.mackintosh@dundee.ac.uk, schen6@163.com or chenshuai@nicemice.cn).

© 2013 The Author(s)
The author(s) has paid for this article to be freely available under the terms of the Creative Commons Attribution Licence (CC-BY) (http://creativecommons.org/licenses/by/3.0/) which permits unrestricted use, distribution and reproduction in any medium, provided the original work is properly cited.

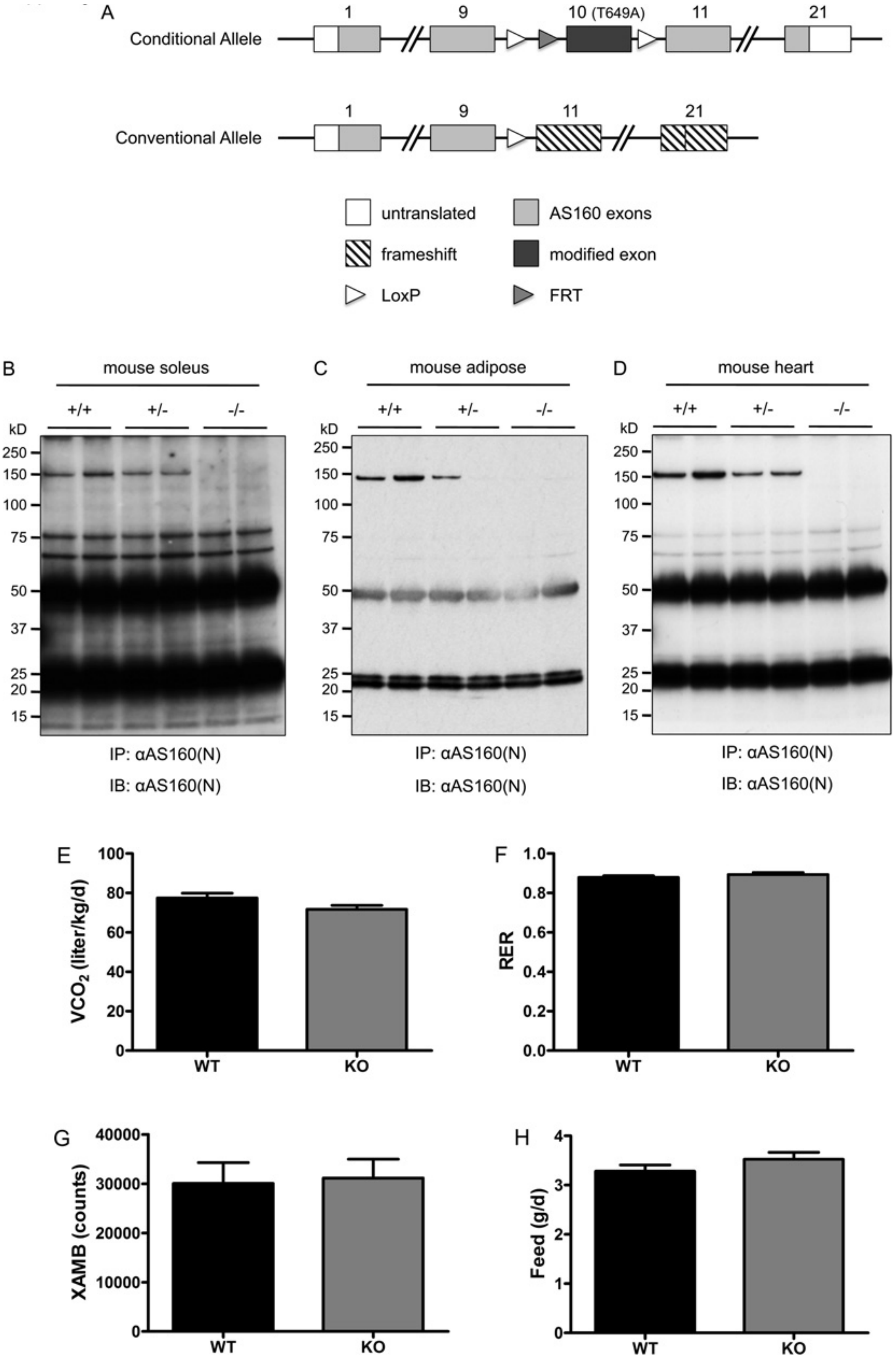


**Figure S1 Generation and basic characterization of the AS160-knockout mouse**

(**A**) Strategy for generating the AS160-knockout mouse. The diagram shows the conditional and conventional allele of AS160. The tenth exon harbouring the T649A mutation is flanked by loxP sites. The AS160-knockout mouse was generated by mating the $AS160^{T649A}$ knockin mouse with the Bal1 mouse line that expresses Cre recombinase in all tissues. The tenth exon harbouring the T649A mutation was excised through loxP-Cre recombination. (**B**–**D**) Immunoprecipitation of full-length AS160 and truncated $AS160^{1-609}$ from tissue lysates. Tissues were removed from 8-week-old male mice. Full-length AS160 proteins and truncated $AS160^{1-609}$ fragment (if the latter had been present) were immunoprecipitated from 60 $\mu$g of soleus lysates, 400 $\mu$g of adipose lysates or 400 $\mu$g of heart lysates using the anti-AS160(N) antibody. The immunoprecipitates were analysed via Western blotting with the anti-AS160(N) antibody (used at 1 $\mu$g/ml at 4°C overnight). The molecular

© 2013 The Author(s)
The author(s) has paid for this article to be freely available under the terms of the Creative Commons Attribution Licence (CC-BY) (http://creativecommons.org/licenses/by/3.0/) which permits unrestricted use, distribution and reproduction in any medium, provided the original work is properly cited.

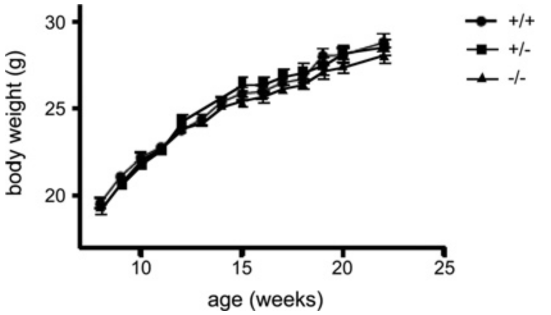


**Figure S2 Body weight of the AS160-knockout mice**

Wild-type (+/+), heterozygous (+/−) and homozygous (−/−) AS160-knockout male mice were weighed once per week from 7 to 23 weeks of age.

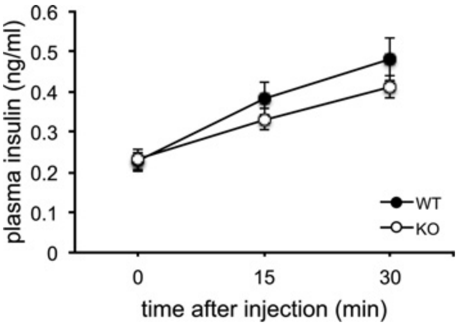


**Figure S3 Plasma insulin levels during a glucose tolerance test**

The data are given as the mean (±S.E.M.) from seven to eight male mice (12–13 weeks old). KO, knockout; WT, wild-type.

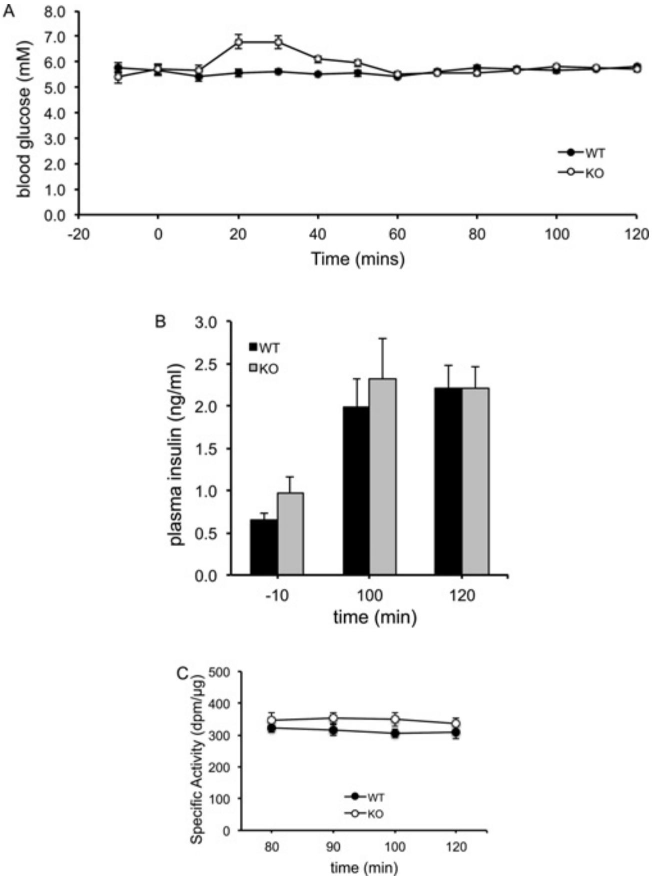


**Figure S4 Blood glucose and plasma insulin levels during hyperinsulinaemic–euglycaemic clamp**

(**A**) Blood glucose levels during euglycaemic clamp in the AS160-knockout and wild-type male mice (16 weeks old). Data are given as means ± S.E.M. for six (wild-type) or ten (knockout) mice. (**B**) Plasma insulin levels before and after hyperinsulinaemic–euglycaemic clamps in the AS160-knockout mice and wild-type littermates. Data are given as means ± S.E.M. for six (wild-type) or ten (knockout) mice. (**C**) Plasma glucose specific activity during $t = 80$ to 120 min of the hyperinsulinaemic–euglycaemic clamp period. The slope of the relationship between glucose specific activity and time was not significantly different from 0, demonstrating that this variable was in a steady state. Data are given as means ± S.E.M. for six (wild-type) or ten (knockout) mice. KO, knockout; WT, wild-type.

mass in kDa is indicated on the left-hand side. (**E**–**H**) Indirect calorimetry measurement. The AS160-knockout and wild-type male mice (8-week-old) were monitored using an Oxymax/CLAMS system (Columbus Instruments). The data are given as the means ± S.E.M. ($n = 6$–7) for $\dot{V}CO_2$ ($CO_2$ production) RER, $X_{AMB}$ (ambulatory activity) and feed intake, as indicated. IB, immunoblot; IP, immunoprecipitate; KO, knockout; WT, wild-type.

© 2013 The Author(s)
The author(s) has paid for this article to be freely available under the terms of the Creative Commons Attribution Licence (CC-BY) (http://creativecommons.org/licenses/by/3.0/) which permits unrestricted use, distribution and reproduction in any medium, provided the original work is properly cited.

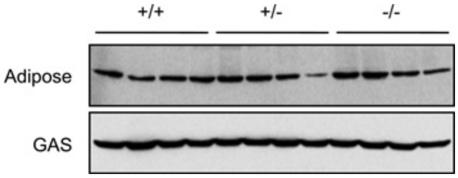


**Figure S5 GLUT1 protein levels in adipose tissue and skeletal muscle**

GLUT1 proteins were detected in 40 $\mu$g of total lysates in adipose tissue and skeletal muscle [gastrocnemius (GAS)] from the AS160-knockout and wild-type mice (8 weeks old) using an anti-GLUT1 antibody.

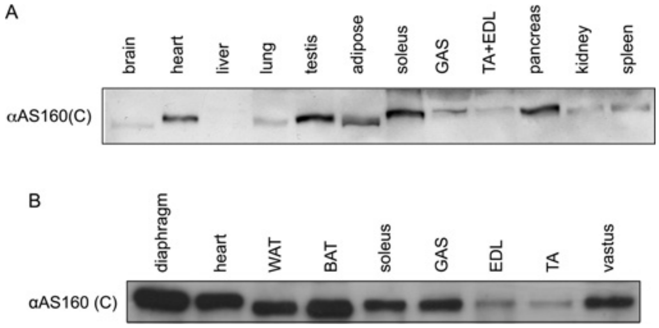


**Figure S6 The expression pattern of AS160 in mouse tissues**

The expression of AS160 was determined in tissues from 8-week-old male wild-type mice. Tissue lysates (40 $\mu$g) were subjected to Western blotting analysis, and AS160 was detected using the anti-AS160(C) antibody. GAS, gastrocnemius.

Received 27 April 2012/2 October 2012; accepted 19 October 2012
Published as BJ Immediate Publication 19 October 2012, doi:10.1042/BJ20120702

© 2013 The Author(s)
The author(s) has paid for this article to be freely available under the terms of the Creative Commons Attribution Licence (CC-BY) (http://creativecommons.org/licenses/by/3.0/) which permits unrestricted use, distribution and reproduction in any medium, provided the original work is properly cited.
